# Protumorigenic Interferon-Stimulated Genes in Cancer: A Comprehensive Review

**DOI:** 10.7759/cureus.63216

**Published:** 2024-06-26

**Authors:** Danial Qasim Butt, Masitah Hayati Harun, Nur Asyilla Che Jalil, Shazana Hilda Shamsuddin, Saidi Jaafar, Basaruddin Ahmad

**Affiliations:** 1 Oral Medicine and Oral Pathology Unit, School of Dental Sciences, Universiti Sains Malaysia, Kubang Kerian, MYS; 2 Pathology, School of Medical Sciences, Universiti Sains Malaysia, Kubang Kerian, MYS; 3 Basic Sciences Unit, School of Dental Sciences, Universiti Sains Malaysia, Kubang Kerian, MYS; 4 Biostatistics Unit, School of Dental Sciences, Universiti Sains Malaysia, Kubang Kerian, MYS

**Keywords:** immunosurveillance, isg, protumorigenic, apoptosis, cancer

## Abstract

Interferon-stimulated genes (ISGs), whose production is triggered by interferons, are known to defend the host from pathogenic and cancer-specific antigens, one of which is by inducing apoptosis in infected or mutated cells. It has been reported recently that specific ISGs aid cancer cells in evading immunosurveillance and inflammatory cells by inhibiting the apoptosis process. This report reviewed four apoptosis-regulating ISG proteins: interferon-stimulated gene 15 (ISG15), interferon alpha-inducible protein 27 (IFI27), interferon alpha-inducible protein 6 (IFI6), and radical S-adenosyl methionine domain containing 2 (RSAD2), demonstrating anti-apoptosis function, and considered them protumorigenic.

## Introduction and background

Interferon-stimulated genes (ISGs) were first discovered as transcriptional genes produced by interferons between late 1979 to 1984 when researchers were investigating methods to trigger interferon production in fibroblast cells as a potential treatment for viral infections [1]. The characterization of numerous ISGs evolved gradually, and their functional roles were discovered between 1985 and 1987 [2]. ISGs are produced by immune cells that are macrophages, dendritic cells, T-cells, neutrophils, plasma cells, natural killer cells, and other connective tissues, including vascular endothelial cells and fibroblasts, and regulated by specific interferons. ISGs are part of innate immunity and have a role in inducing the death of virally infected cells [3]. The ISG transcription is induced by interferons, which are proteins in the family of cytokines produced by dendritic cells to activate host defence [4,5].

ISGs induced by type 1 interferons (IFN-1) included the IFI6, IFI27, interferon-induced protein with tetratricopeptide repeats (IFIT), ISG15, 2'-5'-oligoadenylate synthetase like (OASL), RSAD2, and myxovirus resistance 1 (MX1) genes that are involved in cell apoptosis, cell growth regulation, and angiostatic effects mediation. For types 2 and 3 (IFN-2 and IFN-3), the C-X-C motif chemokine ligand 10 (CXCL10), interferon regulatory factor 8 (IRF8), indoleamine 2,3‐dioxygenase 1 (IDO1), interferon regulatory factor 1 (IRF1), interleukin 10 receptor subunit beta (IL-10RB), and interferon-induced protein 44-like (IFI44L) genes are produced in response to inflammation and autoimmune pathologies [6-8].

Recent reports suggest that some ISGs have roles that function against defence mechanisms and exhibit anti-defensive characteristics that promote tumour progression. Studies on the tumour microenvironment (TME) of breast and colorectal cancer, which comprises cancerous and non-cancerous cells, including fibroblasts, immune cells, blood vessel-forming cells, and proteins, have shown that the upregulation of ISGs is associated with the inhibition of apoptosis and depletion of CD8 T-cells, which are crucial for eliminating cancer cells [9-11].

The change in the role of ISGs from defensive to anti-defensive is likely the result of transmutation following epigenetic reprogramming and immunoediting. This review focuses on the ISGs involved in the apoptosis process to promote tumour progression.

## Review

ISGs and apoptotic regulation

**Figure 1:**
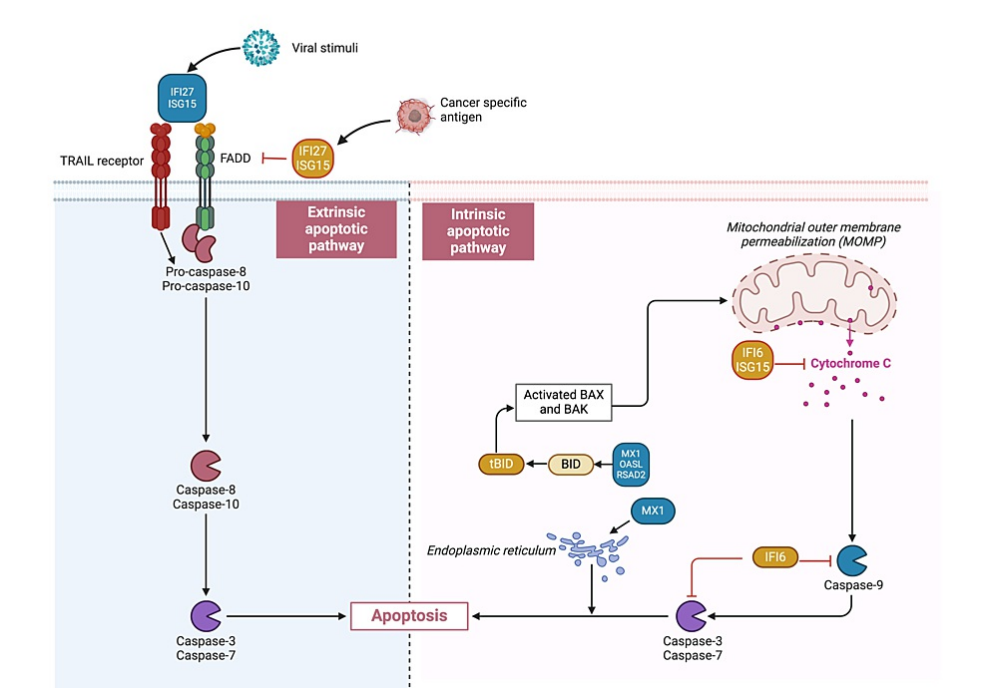
ISGs regulating apoptotic pathways TRAIL: tumour necrosis factor related-spoptosis-inducing ligand; FADD: Fas-associated protein with death domain; tBID: truncated active BID fragment; BID: BH3 interacting-domain death agonist; BAX: Bcl2 like protein 4; BAK: BRI1-associated receptor kinase 1 The image was created with BioRender.com.

Apoptosis is a programmed cell death regulated by the immune system to maintain normal tissue homeostasis and body defence mechanisms in the event of cellular injury or pathogenic exposure. Cell self-destruction via apoptosis involves the caspase cascade pathway and leads to proteolytic degradation/condensation of the nucleus and cytoplasm, which results in cell death. ISGs aid the process in both virally infected and cancer cells via three caspase-mediated pathways: the extrinsic, intrinsic, and endoplasmic reticulum (ER) stress pathways (Figure 1) [12,13].

Extrinsic Pathway

The extrinsic pathway is initiated when pathogens stimulate IFN-1 production, triggering the transcription of ISGs such as ISG15 and IFI27. These ISGs enhance the recruitment and activation of FADD. FADD is an adaptor molecule that transmits apoptotic signals from death receptors on the cell surface, such as the TRAIL receptors to initiate the caspase cascade [14-16].

Intrinsic Pathway

The intrinsic pathway is initiated through contribution by a set of ISGs, such as MX1, CXCL-10, and RSAD2, through different mechanisms. They are found to be assisting in truncating BID in the cytoplasm. The truncated BID then activates BAX and BAK to increase the mitochondrial membrane permeability and release of cytochrome c; this then triggers caspase-9 to activate the caspase cascade within the nucleus [17-19].

ER Stress Pathway

The ER stress pathway is initiated when misfolded proteins accumulate in the ER, leading to the activation of inositol-requiring enzyme-1 (IRE1) on the ER membrane. IRE1 activation can trigger downstream signalling events that include the activation of the caspase-3 cascade, inducing apoptosis in the cell during pathological conditions. However, MX1 in a prostate cancer study was found to be upregulated in ER stress pathway binding with heme-oxygenase (HO) as a downstream target in causing apoptosis in cancer cells [20].

In the initial phases of cancer development, interferons initiate the defence mechanism against cancer by inducing ISG transcription, activating the pathway proteins caspases 3 and 7, and triggering the apoptosis process [21,22]. A few ISGs have been linked to the latter, such as IFI27, IFIT, ISG15 and MX1 in breast colorectal, cervical and prostate cancers, respectively [23-26]. However, recent evidence suggests that ISGs also have a role in promoting tumour growth [27].

Tumour-promoting ISGs through apoptosis inhibition

A review of the literature found several reports claiming that ISGs promote tumorigenesis by facilitating cancer cell growth, progression, metastasis, migration, and invasion. The studies, which investigated immunosuppression, cell growth regulation, angiostatic effects mediation, epithelial-mesenchymal transition, and glycolysis alteration in cancer cells, showed that ISGs are overexpressed in breast, colorectal, pancreatic, oral, oesophagal, stomach, and liver cancers (Table 1).

**Table 1:** Roles of ISGs in cancer ISGs: interferon-stimulated genes; FFPE: formalin fixed paraffin embedded; AKT: protein kinase B; NF-κB: nuclear factor kappa-light chain-enhancer of activated B-cells; PHMG-P: polyhexamethylene guanidine phosphate; mTORC1: mammalian target of rapamycin complex 1; ATM: ataxia-telangiectasia mutated; PABPC-1: poly A binding protein cytoplasmic 1; VEGF-A: vascular endothelial growth factor-A; CRM1/XPO1: chromosomal region maintenance 1/Exportin 1; Wnt-β-catenin: Wingless type 1; JAK: Janus kinase; STAT: signal transducer and activator of transcription proteins; CXCR3: chemokine receptor with 3 ligands; EGFR: epidermal growth factor receptor; ATF3: cyclic AMP-dependent transcription factor 3

Cancer	Experiment/analysis	Mode of action	Outcome	ISG	References
Breast cancer	MDA-MB-231 subclone D3H2LN, Cos1 and HEK293t cell lines	ISGylation, increase in EGFR recycling, and Akt signalling	Tumour progression	ISG15	[28]
Colorectal cancer	LS 174T cell line and FFPE tissue	L1 gene-mediated NF-κB signalling	Tumour progression and metastasis		[29]
Prostate cancer	LNCaP, PC-3 and 22RV-1 cell lines and FFPE tissue	Overexpression under hypoxic conditions	Tumour migration and invasion		[30]
Lung cancer	HPAEpiC cell line	Positive association of PHMG-P with an increase in expression of MX1	Tumour formation	MX1	[31]
Colorectal cancer	FFPE tissue	Positive expression with lymph node metastasis in end-stage patients	Prognostic indicator		[32]
Pancreatic cancer	Panc1, Mia paca 2, ASPC-1 cell lines and FFPE tissue	Increase in expression in cancer tissues in end-stage clinical patients	Tumour invasion and metastasis	OASL	[33]
Breast cancer	Bioinformatics	The increase in mRNA expression in the high/low-risk overall survival groups was the opposite in high/low-risk-free survival groups and increase in neutrophils.	Recurrence and tumour metastasis		[34]
Gastric cancer	GES-1 and STAD cell lines	Regulating mTORC signaling pathway	Tumour progression		[35]
Oral cancer	HEp-2	Increase in expression of OASL in oral cancer tissue with negative association with ATM	Biomarker		[36]
Oral squamous cell carcinoma	TSCCA and TCA8113 cell line, FFPE tissue	Increase in expression in OSCC tissues and cells	Tumour invasion and inhibition of apoptosis	IFI27	[37]
Esophageal squamous cell carcinoma	KYSE150, KYSE520, KYSE510, KYSE410, KYSE450, KYSE180, 81 T, and TE1 cell lines	PABPC1-induced stabilization of IFI27 mRNA	Tumour progression and poor prognosis		[38]
Pancreatic cancer	Bioinformatic analysis	Alteration in glycolysis decreases CD8 T-cells	Tumour progression		[39]
Cholangiocarcinoma	SNU308 cell line	Increase in VEGF-A	Angiogenesis and tumour proliferation		[40]
Breast cancer	MCF-7 cell lines	Regulating ER-α by interacting with CRM1/XPO1	Tumour growth and proliferation		[41]
Hepatocellular and gastric cancer	LH86, Huh7, HLCZ01, HLCZ02, HGC-27 and BGC-823 cell lines	Inhibited TRAIL-induced apoptosis	Tumour formation		[42]
Pancreatic cancer	AsPC-1, MiaPaCa-2, BxPC-3, Patu8988, Panc-1 and CFPAC-1 cell lines	Wnt/β-catenin pathway promotes EMT.	Tumour proliferation and metastasis	IFIT	[43]
Acute myeloid leukemia	Gene expression analysis in cancer tissues	JAK/STAT pathway	Tumour invasion		[44]
Breast cancer	MDA-MB-231 and MDA-MB-468 cell lines	Overexpression of CXCL10 and cross-talk mechanism between CXCR3 and EGFR receptors	Tumour cell migration and invasion	CXCL10	[45]
Esophageal squamous cell carcinoma	Eca109, TE-1, Ec9706, Kyse150, and Kyse410 cell lines and ESCC FFPE tissue	Higher expression correlation with poor prognosis and inhibits apoptosis by reactive oxygen species accumulation	Tumour progression and metastasis	IFI6	[46]
Tongue squamous cell carcinoma	Tongue FFPE cancer tissue and Cal 27, SCC-9, SCC-25 and SCC-4 cell lines	IFI6 overexpressed in 6 tissue samples negatively correlated with ATF3 and inhibited apoptosis in cancer cells	Tumour progression and anti-apoptosis		[47]
Breast cancer	BT-549 and MCF-7	IFI6 induced mitochondrial redox deregulation in breast cancer cells, inhibited apoptosis	Tumour metastasis		[48]
Gastric cancer, lung cancer and breast cancer	FFPE cancer tissue	Higher expression in cancerous tissue of advanced stages had shorter disease-free survival than patients with lower expression in cancerous tissues.	Prognostic indicator	RSAD2	[49]

The reports claimed that some ISGs including IFI6, IFI27 and RSAD2 showed anti-apoptotic roles by directly inhibiting the apoptosis pathway such as arresting the cell cycle leading to inhibition of apoptosis [27,50,51]. For example, IFI6 is shown to directly inhibit the TRAIL-induced extrinsic apoptotic pathway [52], whereas MX1 activates PHMG-P to indirectly block the apoptotic process [31].

ISGs found in cancer cells have been reported to dysregulate specific cytokine signal pathways like JAK/STAT pathways, causing the activation of STAT protein and the activation of anti-apoptotic protein Bcl-2 (Table 1). The Bcl-2 activation causes inhibition of the intrinsic apoptotic pathway [44].

In the present review, four ISGs are found to directly interfere with the apoptotic pathway: ISG15, IFI27, IFI6, and RSAD2 and focus on the current understanding of them.

ISG15 is a member of the ubiquitin-like protein family and is involved in many cellular processes, including immune regulation, autophagy, and cancer progression. It has been shown to exhibit complex dual roles of promoting and suppressing tumour growth and metastasis in different tumour systems by promoting cancer growth and inhibiting cancer cell apoptosis [53]. Studies on breast cancer found that proteins undergoing post-translational modification by ISGylation sustain Akt signalling, which inhibits the caspase-mediated apoptotic pathways and blocks cancer cell destruction [28]. In prostate cancer, overexpression of ISG15 is regulated by hypoxia-inducible factors (HIF) under hypoxic conditions promoting the release of the anti-apoptotic protein Bcl2, which inhibits apoptosis [30]. In colorectal cancer studies, ISG15 altered NF-κB signalling by conjugating to key signalling proteins such as IκBα (inhibitor of κB alpha) and p65 (a subunit of NF-κB), thereby causing inhibition of apoptosis [29].

IFI27, also known as ISG12 or p27, belongs to the IFN-inducible genes. IFI27 downregulation and upregulation in oral squamous cell carcinoma (OSCC) cell lines are linked to an increase and decrease in the percentage of apoptotic cancer cells, respectively, indicating that an upregulation increases cancer cell survival [37]. Only the OSCC study has connected IFI27 to promoting tumorigenesis by interfering with apoptosis. The metabolic regulation mediated by IFI27 causes glycolysis deregulation in CD8 T-cells, decreases its number, and aids cancer cells to progress [39]. IFI27 has been reported to be involved in the growth of blood vessels by upregulating vascular endothelial growth factor-A (VEGF-A) in cancer cells [40].

IFI6, also known as IFN alpha inducible protein 6 and G1P3, belongs to the FAM14 family genes and is found to inhibit apoptosis in various cancer systems. In human multiple myeloma cell lines, IF16 temporarily antagonises the TRAIL-induced apoptosis by preserving the mitochondrial integrity, such as Bcl-2 family proteins, thereby preventing the release of pro-apoptotic factors and inhibiting the TRAIL-induced caspase cleavage via death-inducing signalling complexes (DISC) [46]. Overexpression of IFI6 in breast cancer cells inhibits apoptosis by inhibiting the tumour-suppressing IFN-1 characteristics, activating immune-endocrine-elicited redox signalling [48]. This later interferes with mtROS-mediated apoptosis by blunting the mitochondrial permeability and preventing the toxicity of high mtROS from inducing apoptotic pathways [46]. Suppressing IFI6 in oesophageal squamous cell carcinoma cell lines elevates calcium uptake by mitochondria, accumulates reactive oxygen species, and induces apoptosis. Conversely, when IFI6 is ectopically expressed, the mitochondrial membrane potential is deregulated, inhibiting caspase-mediated apoptosis [46]. In breast cancer, upregulation of IFI6 in endosomes and mitochondria and the binding IFI6 with RAB+ endosomes dysregulate the mitochondrial resistance to apoptosis and thus ensued anti-apoptosis in cancer cells [48].

Radical S-adenosyl methionine domain containing 2 (RSAD2) is an antiviral protein from the S-adenosyl-L-methionine (SAM) superfamily of viperin enzymes. Only one report claimed it promotes tumorigenesis by interfering with the apoptosis process. An immunohistochemistry study found that RSAD2 was highly expressed in cancerous compared to non-cancerous areas of tissue samples of gastric, lung, and breast cancer. Advanced clinical stage patients with higher RSAD2 expression, compared to those with low expression, were also found to have a shorter disease-free survival period [48].

Protumorigenic ISGs

ISGs have been previously linked to a defensive role in viral infection and early cancer development studies [54]. Emerging evidence suggests that they also demonstrate tumour-promoting characteristics, one of which is inhibiting apoptosis of cancer cells, allowing them to continue to proliferate and progress.

The behaviour of the four ISGs above demonstrate characteristics that are consistent with the term protumorigenic, which was first referred to by Nguyen et al. (2023) as proteins or genes in cancer tissues that undergo epigenetic modification, with or without mutations, and have developed the capability to promote tumour progression and metastasis; in this context, by ensuring the survival of cancer cells through inhibition of programmed cell death [55,56]. Authors in cancer studies referred to the tumour-promoting effects of IFN-1 as protumorigenic interferon alpha receptor 1 (IFNAR1) [10,51]. Thus, it is only sensible to refer to the four ISGs as protumorigenic ISGs based on their roles in promoting tumorigenesis and to discriminate them from those having a defensive role.

There is currently little discussion on how the roles of ISGs changed to become protumorigenic but because ISGs are induced by interferons, the modification is likely instigated by protumorigenic interferons which are responsible for modulating the extrinsic and intrinsic apoptosis responses and the production and mode of action/nature of ISG [57].

According to Musella et al. (2022), protumorigenic interferons are the product of epigenetic reprogramming by dormant cancer cells [10]. Epigenetic reprogramming alters the phenotypic characteristics of cytokines through DNA methylation and chromatin remodelling but does not change the DNA sequence [58]. During the elimination phase of tumourigenesis, cancer-specific antigens prompt the production of interferons to trigger the transcription of ISGS to eliminate cancer cells, one of which is via apoptosis [59]. During the equilibrium phase, some unstable dormant cancer cells in the TME survive the immunosurveillance and aided by upregulated epigenetic factor lysine (K)-demethylase 1B (KDM1B), the cytokines/interferons undergo epigenetic remodelling to yield protumorigenic IFN-1. The result of the process is a new epigenetically reprogrammed cancer cell with enhanced aggressiveness and with capability of producing protumorigenic ISGs [10].

TME also contributes to epigenetic reprogramming as the extracellular matrix is rich in stromal and immune cells that produce cytokines and chemokines and foster a chronic inflammatory state that promotes tumorigenesis [57]. Research has demonstrated that ISGs released by cancer cells into the TME inhibit the anti-tumour function of cytotoxic T-lymphocytes (CTLs) through the activation of metabolic enzymes such as IDO1, the activation of the inhibitory receptor, programmed cell death protein 1 (PD-1) by its major ligand, programmed cell death ligand 1 (PD-L1), and extrinsic suppression by Forkhead box P3 (FoxP3+) and regulatory T cells (Tregs) [60,61]. Suppression of immunosurveillance by IFN-1 in TME converts mature anti-tumour neutrophils and macrophages to immature tumour-associated neutrophils N2 (TAN N2), and macrophages M2 (TAM M2) phenotypes following an imbalance in the STAT activation pathways [62]. The TAN (N2) and TAM (M2) cause an immunosuppressive microenvironment by releasing chemokines to prevent CTL proliferation and inhibiting the capability for recognising cancer antigens through blocking of its T-cell receptors [63,64]. Similarly, studies on plasma cells in TME demonstrated plasma cells' polarisation to tumour-associated plasma cells. These tumour-associated plasma cells produce IgG that inhibits macrophages and lymphocytes anti-tumour function involved in the cross-talk between the cancer cells helping cancer cells to immune escape [65,66].

**Figure 2:**
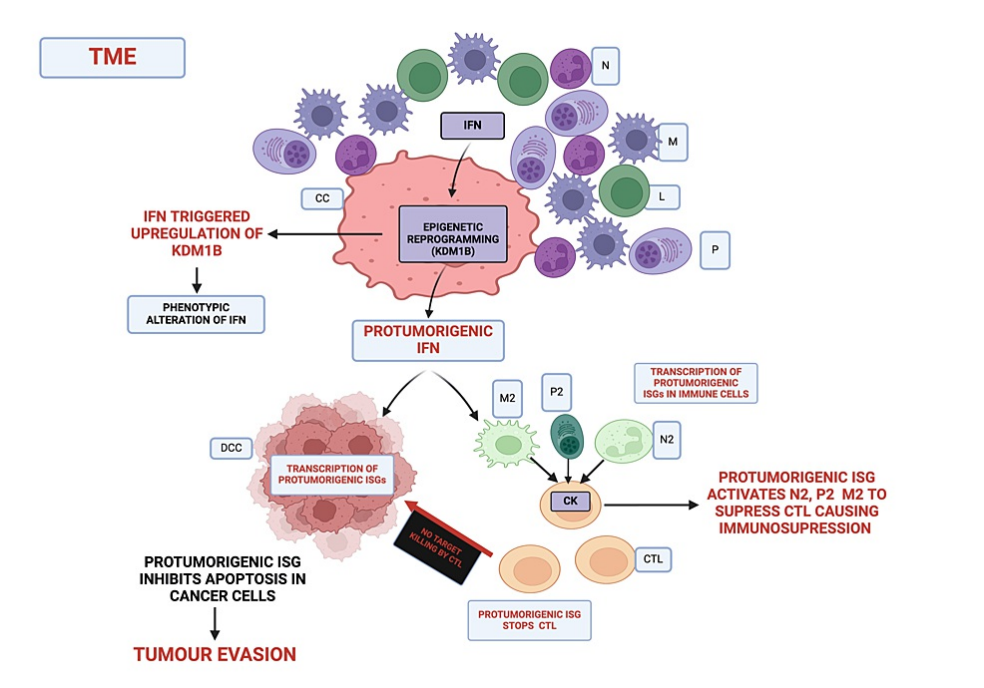
Hypothetical view of protumorigenic ISG production and role in TME ISG: interferon-stimulated gene; TME: tumour microenvironment; N: neutrophil; M: macrophage; L: lymphocyte; P: plasma cell; CC: cancer cell; DCC: dormant cancer cell; N2: immature neutrophil-tumour associated; M2: immature macrophage-tumour associated; P2: polarised plasma cell-tumour associated; CK: chemokines The figure was created with BioRender.com.

Based on the findings from Musella et al. and studies on the polarisation of immune cells in TME, it is understood that during immunosurveillance, the interferons in TME trigger upregulation of KDM1B in dormant cancer cells, which, in turn, remodels the former through immunoediting, changing its phenotype characteristics to protumorigenic. This protumorigenic interferon then triggers the JAK/STAT pathway to produce protumorigenic ISGs that inhibit caspase-mediated apoptosis and render cancer cells with stemness-like properties. It can be speculated that the protumorigenic IFN also leaves the cell a paracrine effect and binds to IFNAR and induces similar protumorigenic ISGs in the nearby dormant cancer cells. It also infiltrates other immune cells in TME by inhibiting cytotoxic T-cells directly and/or triggering the immature neutrophils (N2), polarised plasma cells (P2), and immature macrophages (M2) to repress the cytotoxic T-cells’ function of eliminating the cancer cells (Figure 2).

## Conclusions

Studies indicate that there is a distinction between protumorigenic ISGs and defensive ISGs and that the former, found in TME, are either epigenetically reprogrammed or genetically mutated to aid in cancer cell proliferation and progression by, among others, inhibiting apoptosis and suppressing immune surveillance.

Protumorigenic ISGs have been found in many cancer cells but have not been studied in the microenvironment together. Among the protumorigenic ISGs involved in apoptosis, two, the protumorigenic IFI6 and RSAD2, in OSCC TME, are less studied. Further, it is pertinent to discriminate between protumorigenic and defensive ISG variants in TME at tissue and molecular levels to better understand their involvement in the apoptosis process and apply them in immunotherapy.

## References

[1] Larner AC, Jonak G, Cheng YS, Korant B, Knight E, Darnell JE Jr (1984). Transcriptional induction of two genes in human cells by beta interferon. Proc Natl Acad Sci U S A.

[2] Haas AL, Ahrens P, Bright PM, Ankel H (1987). Interferon induces a 15-kilodalton protein exhibiting marked homology to ubiquitin. J Biol Chem.

[3] Schoggins JW, Rice CM (2011). Interferon-stimulated genes and their antiviral effector functions. Curr Opin Virol.

[4] Ali S, Mann-Nüttel R, Schulze A, Richter L, Alferink J, Scheu S (2019). Sources of type I interferons in infectious immunity: plasmacytoid dendritic cells not always in the driver’s seat. Front Immunol.

[5] Boukhaled GM, Harding S, Brooks DG (2021). Opposing roles of type I interferons in cancer immunity. Annu Rev Pathol.

[6] Avraham EM, Carmi VD, Alteber Z, Farago AM, Tzehoval E, Eisenbach L (2013). The human ISG12a gene is a novel caspase dependent and p53 independent pro-apoptotic gene, that is overexpressed in breast cancer. Cell Biol Int Rep.

[7] Khabar KS, Al-Haj L, Al-Zoghaibi F, Marie M, Dhalla M, Polyak SJ, Williams BR (2004). Expressed gene clusters associated with cellular sensitivity and resistance towards anti-viral and anti-proliferative actions of interferon. J Mol Biol.

[8] Solis M, Goubau D, Romieu-Mourez R, Genin P, Civas A, Hiscott J (2006). Distinct functions of IRF-3 and IRF-7 in IFN-alpha gene regulation and control of anti-tumor activity in primary macrophages. Biochem Pharmacol.

[9] Koelzer VH, Sokol L, Zahnd S (2017). Digital analysis and epigenetic regulation of the signature of rejection in colorectal cancer. Oncoimmunology.

[10] Musella M, Guarracino A, Manduca N (2022). Type I IFNs promote cancer cell stemness by triggering the epigenetic regulator KDM1B. Nat Immunol.

[11] Yang L, Dong X, Liu Z (2022). VPS9D1-AS1 overexpression amplifies intratumoral TGF-β signaling and promotes tumor cell escape from CD8(+) T cell killing in colorectal cancer. Elife.

[12] Jan R, Chaudhry GE (2019). Understanding apoptosis and apoptotic pathways targeted cancer therapeutics. Adv Pharm Bull.

[13] Xin XL, Zhang R, Yuan XM, Liu L (2019). Mechanisms of IFNalpha-1A-induced apoptosis in a laryngeal cancer cell line. Med Sci Monit.

[14] Ganesan M, New-Aaron M, Dagur RS (2019). Alcohol metabolism potentiates HIV-induced hepatotoxicity: contribution to end-stage liver disease. Biomolecules.

[15] Xie F, Zhang Y, Li J (2022). MiR-942-5p targeting the IFI27 gene regulates HCT-8 cell apoptosis via a TRAIL-dependent pathway during the early phase of Cryptosporidium parvum infection. Parasit Vectors.

[16] Zhou H, Zhang Y, Wang J (2022). The CREB and AP-1-dependent cell communication network factor 1 regulates porcine epidemic diarrhea virus-induced cell apoptosis inhibiting virus replication through the p53 pathway. Front Microbiol.

[17] Hardy S, Jackson B, Goodbourn S, Seago J (2020). Classical swine fever virus N(pro) antagonises IRF3 to prevent IFN-independent TLR3 and RIG-I-mediated apoptosis. J Virol.

[18] Sarkar R, Nandi S, Lo M, Gope A, Chawla-Sarkar M (2021). Viperin, an IFN-stimulated protein, delays rotavirus release by inhibiting non-structural protein 4 (NSP4)-induced intrinsic apoptosis. Viruses.

[19] Teymoori-Rad M, Shokri F, Salimi V, Marashi SM (2019). The interplay between vitamin D and viral infections. Rev Med Virol.

[20] Ortiz E, Sanchis P, Bizzotto J (2020). Myxovirus resistance protein 1 (MX1), a novel HO-1 interactor, tilts the balance of endoplasmic reticulum stress towards pro-death events in prostate cancer. Biomolecules.

[21] Chawla-Sarkar M, Lindner DJ, Liu YF, Williams BR, Sen GC, Silverman RH, Borden EC (2003). Apoptosis and interferons: role of interferon-stimulated genes as mediators of apoptosis. Apoptosis.

[22] Shi W, Yao X, Fu Y, Wang Y (2022). Interferon-α and its effects on cancer cell apoptosis. Oncol Lett.

[23] Diwanji N, Bergmann A (2019). Two sides of the same coin - compensatory proliferation in regeneration and cancer. Adv Exp Med Biol.

[24] Ohsugi T, Yamaguchi K, Zhu C, Ikenoue T, Furukawa Y (2017). Decreased expression of interferon-induced protein 2 (IFIT2) by Wnt/β-catenin signaling confers anti-apoptotic properties to colorectal cancer cells. Oncotarget.

[25] Sanwlani R, Kang T, Gummadi S, Nedeva C, Ang CS, Mathivanan S (2023). Bovine milk-derived extracellular vesicles enhance doxorubicin sensitivity in triple negative breast cancer cells by targeting metabolism and STAT signalling. Proteomics.

[26] Zhou MJ, Chen FZ, Chen HC, Wan XX, Zhou X, Fang Q, Zhang DZ (2017). ISG15 inhibits cancer cell growth and promotes apoptosis. Int J Mol Med.

[27] Li J, Ko JM, Dai W, Yu VZ, Ng HY, Hoffmann JS, Lung ML (2021). Depletion of DNA polymerase theta inhibits tumor growth and promotes genome instability through the cGAS-STING-ISG pathway in esophageal squamous cell carcinoma. Cancers (Basel).

[28] Bolado-Carrancio A, Lee M, Ewing A (2021). ISGylation drives basal breast tumour progression by promoting EGFR recycling and Akt signalling. Oncogene.

[29] Cheriyamundath S, Basu S, Haase G, Doernberg H, Gavert N, Brabletz T, Ben-Ze'ev A (2019). ISG15 induction is required during L1-mediated colon cancer progression and metastasis. Oncotarget.

[30] Lyu F, Li Y, Yan Z (2022). Identification of ISG15 and ZFP36 as novel hypoxia- and immune-related gene signatures contributing to a new perspective for the treatment of prostate cancer by bioinformatics and experimental verification. J Transl Med.

[31] Lee H, Jeong SH, Lee H (2022). Analysis of lung cancer-related genetic changes in long-term and low-dose polyhexamethylene guanidine phosphate (PHMG-p) treated human pulmonary alveolar epithelial cells. BMC Pharmacol Toxicol.

[32] Croner RS, Stürzl M, Rau TT (2014). Quantitative proteome profiling of lymph node-positive vs. -negative colorectal carcinomas pinpoints MX1 as a marker for lymph node metastasis. Int J Cancer.

[33] Chen S, Sun Z, Zhao W (2022). Oligoadenylate synthetases-like is a prognostic biomarker and therapeutic target in pancreatic ductal adenocarcinoma. Ann Transl Med.

[34] Zhang Y, Yu C (2020). Prognostic characterization of OAS1/OAS2/OAS3/OASL in breast cancer. BMC Cancer.

[35] Zhao W, Yang H, Liu L (2023). OASL knockdown inhibits the progression of stomach adenocarcinoma by regulating the mTORC1 signaling pathway. FASEB J.

[36] Huang CH, Huang YC, Xu JK, Chen SY, Tseng LC, Huang JL, Lin CS (2023). ATM inhibition-induced ISG15/IFI27/OASL is correlated with immunotherapy response and inflamed immunophenotype. Cells.

[37] Wang H, Qiu X, Lin S, Chen X, Wang T, Liao T (2018). Knockdown of IFI27 inhibits cell proliferation and invasion in oral squamous cell carcinoma. World J Surg Oncol.

[38] Zhang Y, Chen C, Liu Z, Guo H, Lu W, Hu W, Lin Z (2022). PABPC1-induced stabilization of IFI27 mRNA promotes angiogenesis and malignant progression in esophageal squamous cell carcinoma through exosomal miRNA-21-5p. J Exp Clin Cancer Res.

[39] Huang S, Zhao J, Song J (2021). Interferon alpha-inducible protein 27 (IFI27) is a prognostic marker for pancreatic cancer based on comprehensive bioinformatics analysis. Bioengineered.

[40] Chiang KC, Huang ST, Wu RC (2019). Interferon α-inducible protein 27 is an oncogene and highly expressed in cholangiocarcinoma patients with poor survival. Cancer Manag Res.

[41] Cervantes-Badillo MG, Paredes-Villa A, Gómez-Romero V (2020). IFI27/ISG12 downregulates estrogen receptor α transactivation by facilitating its interaction with CRM1/XPO1 in breast cancer cells. Front Endocrinol (Lausanne).

[42] Liu N, Zuo C, Wang X, Chen T, Yang D, Wang J, Zhu H (2014). miR-942 decreases TRAIL-induced apoptosis through ISG12a downregulation and is regulated by AKT. Oncotarget.

[43] Li TH, Zhao BB, Qin C (2021). IFIT1 modulates the proliferation, migration and invasion of pancreatic cancer cells via Wnt/β-catenin signaling. Cell Oncol (Dordr).

[44] Zhao Y, Zhang Y, Lu W (2023). The diagnostic/prognostic roles and biological function of the IFIT family members in acute myeloid leukemia. BMC Med Genomics.

[45] Tsutsumi E, Stricklin J, Peterson EA, Schroeder JA, Kim S (2022). Cxcl10 chemokine induces migration of ING4-deficient breast cancer cells via a novel cross talk mechanism between the Cxcr3 and EGFR receptors. Mol Cell Biol.

[46] Liu Z, Gu S, Lu T, Wu K, Li L, Dong C, Zhou Y (2020). IFI6 depletion inhibits esophageal squamous cell carcinoma progression through reactive oxygen species accumulation via mitochondrial dysfunction and endoplasmic reticulum stress. J Exp Clin Cancer Res.

[47] Xu L, Zu T, Li T (2021). ATF3 downmodulates its new targets IFI6 and IFI27 to suppress the growth and migration of tongue squamous cell carcinoma cells. PLoS Genet.

[48] Cheriyath V, Kaur J, Davenport A, Khalel A, Chowdhury N, Gaddipati L (2018). G1P3 (IFI6), a mitochondrial localised antiapoptotic protein, promotes metastatic potential of breast cancer cells through mtROS. Br J Cancer.

[49] Choi KM, Kim JJ, Yoo J (2022). The interferon-inducible protein viperin controls cancer metabolic reprogramming to enhance cancer progression. J Clin Invest.

[50] Wani AK, Akhtar N, Mir TU (2023). Targeting apoptotic pathway of cancer cells with phytochemicals and plant-based nanomaterials. Biomolecules.

[51] Odnokoz O, Yu P, Peck AR (2020). Malignant cell-specific pro-tumorigenic role of type I interferon receptor in breast cancers. Cancer Biol Ther.

[52] Cheriyath V, Glaser KB, Waring JF, Baz R, Hussein MA, Borden EC (2007). G1P3, an IFN-induced survival factor, antagonizes TRAIL-induced apoptosis in human myeloma cells. J Clin Invest.

[53] Yuan Y, Qin H, Li H (2023). The functional roles of ISG15/ISGylation in cancer. Molecules.

[54] Munnur D, Banducci-Karp A, Sanyal S (2022). ISG15 driven cellular responses to virus infection. Biochem Soc Trans.

[55] Kalvakolanu DV (2004). The GRIMs: a new interface between cell death regulation and interferon/retinoid induced growth suppression. Cytokine Growth Factor Rev.

[56] Nguyen HM, Gaikwad S, Oladejo M, Agrawal MY, Srivastava SK, Wood LM (2023). Interferon stimulated gene 15 (ISG15) in cancer: an update. Cancer Lett.

[57] Costoya JA, Arce VM (2023). Cancer cells escape the immune system by increasing stemness through epigenetic reprogramming. Cell Mol Immunol.

[58] Das D, Karthik N, Taneja R (2021). Crosstalk between inflammatory signaling and methylation in cancer. Front Cell Dev Biol.

[59] Musella M, Galassi C, Manduca N, Sistigu A (2021). The yin and yang of type I IFNs in cancer promotion and immune activation. Biology (Basel).

[60] Gajewski TF, Schreiber H, Fu YX (2013). Innate and adaptive immune cells in the tumor microenvironment. Nat Immunol.

[61] Zhang Y, Guan XY, Jiang P (2020). Cytokine and chemokine signals of T-cell exclusion in tumors. Front Immunol.

[62] Pylaeva E, Lang S, Jablonska J (2016). The essential role of type I interferons in differentiation and activation of tumor-associated neutrophils. Front Immunol.

[63] Khanmohammadi S, Rezaei N (2022). Tumor associated neutrophils (TANs) and cancer metastasis. Handbook of Cancer and Immunology.

[64] Gankema AA, Furumaya C, Fernández-Hermira S, Hoogenboezem M, Matlung HL, van Bruggen R, Kuijpers TW (2023). Efficient complement-mediated clearance of immunosuppressed T cells by macrophages. Front Immunol.

[65] Wei Y, Lao XM, Xiao X (2019). Plasma cell polarization to the immunoglobulin G phenotype in hepatocellular carcinomas involves epigenetic alterations and promotes hepatoma progression in mice. Gastroenterology.

[66] Lee HE, Luo L, Kroneman T (2020). Increased plasma cells and decreased B-cells in tumor infiltrating lymphocytes are associated with worse survival in lung adenocarcinomas. J Clin Cell Immunol.

